# *Tridax procumbens* Ameliorates Streptozotocin-Induced Diabetic Neuropathy in Rats via Modulating Angiogenic, Inflammatory, and Oxidative Pathways

**DOI:** 10.1155/2022/1795405

**Published:** 2022-08-31

**Authors:** Munish Kakkar, Tapan Behl, Celia Vargas-De-La Cruz, Hafiz A. Makeen, Mohammed Albratty, Hassan A. Alhazmi, Abdulkarim M. Meraya, Ghadeer M. Albadrani, Mohamed M. Abdel-Daim

**Affiliations:** ^1^Chitkara College of Pharmacy, Chitkara University, Rajpura, Punjab, India; ^2^School of Health Sciences & Technology, University of Petroleum and Energy Studies, Bidholi 248007, Dehradun, Uttarakhand, India; ^3^Department of Pharmacology, Bromatology and Toxicology, Faculty of Pharmacy and Biochemistry, Universidad Nacional Mayor de San Marcos, Lima, Peru; ^4^E-Health Research Center, Universidad de Ciencias y Humanidades, Lima, Peru; ^5^Pharmacy Practice Research Unit, Clinical Pharmacy Department, College of Pharmacy, Jazan University, Jazan, Saudi Arabia; ^6^Department of Pharmaceutical Chemistry and Pharmacognosy, College of Pharmacy, Jazan University, Jazan, Saudi Arabia; ^7^Substance Abuse and Toxicology Research Center, Jazan University, Jazan, Saudi Arabia; ^8^Department of Pharmacy Practice, Faculty of Pharmacy, Jazan University, P.O. Box 114-45124, Jazan, Saudi Arabia; ^9^Department of Biology, College of Science, Princess Nourah bint Abdulrahman University, P.O. Box 84428, Riyadh 11671, Saudi Arabia; ^10^Department of Pharmaceutical Sciences, Pharmacy Program, Batterjee Medical College, P.O. Box 6231, Jeddah 21442, Saudi Arabia; ^11^Pharmacology Department, Faculty of Veterinary Medicine, Suez Canal University, Ismailia 41522, Egypt

## Abstract

*Tridax procumbens* (TP) is a traditional Indian therapeutic plant and was evaluated for its blood glucose lowering abilities, as well as for its ability to curb diabetic neuropathy (DN). Administrating 45 mg/kg body weight of streptozotocin (STZ) intraperitoneally for four weeks, DN was induced in Wistar rats. After the rats' tails were clipped, the blood glucose levels were measured. Body weight and urine volume were also assessed. Oxidative stress makers such as superoxide dismutase (SOD), thiobarbituric acid reactive substances (TBARS), catalase (CAT), inflammatory cytokines for instance tumor necrosis factor (TNF)-*α*, and interleukin (IL)-1*β* were estimated. Further, protein kinase C (PKC-*β*) and vascular endothelial growth factor (VEGF) were also estimated as angiogenic markers. Behavioral parameters were also evaluated by using cold allodynia using acetone test, hot allodynia using Eddy's hot plate, grip strength test using Rota rod, and hyperalgesia test using Tail flick technique. The statistical assessment of findings was done employing one-way (ANOVA) analysis of variance, and subsequently Turkey as *post hoc* with GraphPad Prism software package. The ingestion of TP for 1 month in DN rats stemmed in a substantial decline in blood glucose concentrations matched to nontreated rats with DN. There had been a considerable improvement in DN as evident from the finding from biochemical markers. The serum level of antioxidant defense enzymes was significantly increased, while the activities of TBARS had been substantially reduced in the TP treated rats with DN. TP averted DN-triggered surge levels of TNF-*α* and IL-6 in the serum. Further, PKC-*β* and VEGF concentrations had been also reduced by the treatment TP. The findings of this research demonstrated that the restorative impact of TP on DN rats might be linked to the anti-inflammatory and antioxidative antiangiogenic retorts.

## 1. Introduction

The condition of peripheral neuropathy is extremely complex and prevalent. In the general population, approximately 8% of individuals have peripheral neuropathy, which rises to 15 percent in individuals older than 40 years [1]. Prediabetes and type 2 diabetes (T2D) are the utmost common roots of peripheral neuropathy in the Europe and US. The majority of diabetic patients, together with those with type-1 diabetes (T1D), progress to neuropathy at some point in their lives. The incidence of prediabetes and type-2 diabetes is increasing worldwide, especially in countries that consume more western foods. As more people of America progress to prediabetes and T2D, the number of those with neuropathy will double. Currently, more than twenty million American people have diabetic neuropathy as a result of prediabetes, T1D, or T2D [2]. There are also 316 million people worldwide, who suffer from prediabetes, and 387 million who suffer from diabetes, respectively, and despite a lack of exact figures, the figures suggest that at least 200 million of those with diabetes suffer from neuropathy.

Multiple forms of peripheral nervous system (PNS) harm can be caused by diabetes. A stocking-glove neuropathy is the most common type of nerve damage. This condition has, therefore, become synonymous with diabetic neuropathy (DN). The pattern of injury is similar in those possessing prediabetic conditions, auxiliary to the notion that damage of nerve from diabetes has been related to blood sugar levels ranging from normal to hyperglycemic [3]. A primary characteristic of DN is disordered sensory processing in the feet, which can lead to positive and negative symptoms, including tingling, pain, and tingling sensations (paraesthesia), as well as numbness; discomfort caused by a loss of sensory function may cause pain once touched (allodynia) the feet and increased hyperalgesia (pain sensitivity). Symptoms of motor nerve dysfunction seem far along in the progression of the disease and typically manifest as distal weakness of the toes, calves, and ankles. There is currently no explanation as to where axons-sensory is susceptible to conditions of diabetes more than axons-motor. Over time, lower extremity sensation, as well as motor weakness, led to falls, numb, and insensitive feet.

The only modifiable treatment for DN is improving lifestyle and controlling diabetes, despite decades of research. Based on a review from Cochrane database, which reviewed all the retrieved and available clinical trials, hard glucose control appears to lower the occurrence of diabetic neuropathy in patients with T1D but has very little or no outcome on patients with T2D despite having improved glucose control for more than 10 years [4]. Callaghan et al. suggested that it is not one disease, but two, with alike clinical exhibitions. Clearly, the mechanisms underlying DN differ between T2D and T1D, and this difference is informative. For the last two decades, diabetes mellitus pathogenesis has been studied using glucose and the T1D rat as a model despite this paradox. All United States trials targeting an intervention to alter the progressive nature of DN have botched. Large pharmaceutical companies are now walking away from the disease due to a lack of basic understanding. This causes a high burden on society, but the individual costs are even greater, owing to the ache and helplessness to work laterally with deprived quality of life (QWL) each patient suffers. As a consequence of the enormity of the issue, both at an individual and societal level, mechanisms need to be understood, and early diagnosis to prevent poor patient outcomes is required [5].

It has become more urgent than ever to explore novel ways for treating diabetes and preventing its onset while reducing the side effects of other conventional medications. Despite the fact that conventional therapies are effective and satisfactory, but there is an increasing number of side effects associated with them, developing newfangled medicines sourced from natural sources with lesser adverse effects and similar effectiveness as conventional medicines is urgently needed [6–9]. The popularity of plants is based on their effectiveness, ease of accessibility, low cost, and relative lack of harmful effects. For instance, *Bacopa monnieri* contains compounds that enhance memory, *Curcuma longa* has anti-inflammatory properties, *Momordica charantia* is hypoglycemic, etc. These have shown their respective therapeutic properties without side effects. According to recent reports, WHO strongly recommends Artemisinin and its derivatives from *Artemisia annua* as a treatment for malaria caused by *Plasmodium falciparum*, since synthetic antimalarials alone are ineffective [10]. Similarly, a flavonoid isolated from milk thistle, that is, *Silybum marianum,* has been granted approval as drug for treating various liver disorders in Germany and western countries [10].

*Tridax procumbens* extracts were found to hold anti-inflammatory, analgesic, and antidiabetic functionalities in animal models. Preclinical studies have indicated that acute and subacute administration of *Tridax procumbens* to diabetic (induced by alloxan) rats reduced fasting glucose levels in blood, but not those of control rats without diabetes. In rats loaded with cholesterol (1 g/100 g body weight), *Tridax procumbens* also reduced blood levels of total cholesterol (TC), LDL, VLDL, and triglycerides levels, along with atherogenic index, and atherogenic coefficient [11]. The effectiveness of *Tridax procumbens* in controlling blood glucose and lowering cardiovascular risk in diabetics has not been studied in clinical studies. There has been as well a paucity of evidence concerning the molecular mechanisms that underlie the anti-inflammatory and antihyperglycemic activities of *Tridax procumbens*. In this study, *Tridax procumbens* was evaluated for its blood glucose lowering abilities as well as for its ability to curb diabetic neuropathy. The body weight, urine volume, and glycated hemoglobin were also assessed. Behavioral parameters were also assessed using various *in vivo* models using rats. Further, the resolution of this current investigation was to explore possible mechanisms for antihyperglycemic, anti-inflammatory, angiogenic, and antioxidant effects *in vivo* using various biochemical marker estimations.

## 2. Materials and Methods

### 2.1. The Plant and the Extracts

*Tridax procumbens* whole plant was collected from National Institute of Pharmaceutical Education and Research (NIPER), Mohali, Punjab. The collection has been done during the months of August and September. The voucher specimen was deposited and was stored in a publicly available herbarium of the institute with reference number NIP-H-290. A mechanical grinder was used to chop and pulverize the plant parts after being shade dried. After powdering the crude extract (1 kg), ether was used to defat it. For comprehensive removal of fatty ingredients, this step was repeated three times. The samples were then subjected to a series of fractionations using increasingly polarized organic solvents: methanol, petroleum ether, and ethyl acetate. After comprehensive extraction of the crude drug, the four extracts were received, and under condensed pressure, the extracts were concentrated. Four concentrated extracts of *Tridax procumbens* (TP) were obtained (yield 0.65%, 0.65%, 0.65%, and 0.65% w/w when calculated with respect to the starting dried plant material). After that, the final extracts were cooled to 4°C before use.

### 2.2. Animals

Adult male and female Wister rats (weighing 100–250 g) were employed for this experimentation. The rats were arranged from the animal facility, Chitkara College of Pharmacy, Chitkara University, Punjab. Polypropylene cages have been used for housing the animals (atmosphere controlled at 23 ± 1°C temperature together with a light and dark cycle of 12 h). An acclimatization period of one week preceded the experiment for all animals. In addition to a standard pellet diet, the rats had unrestricted approach to drinking water. By protocol reference number IAEC/CCP/21/02/PR-010, the study protocols have been permitted by the IAEC. During the study, the rats were taken care of and used in compliance with the standards prescribed by Committee for the Purpose of Control and Supervision of Experiments on Animals (CPCSEA), India.

### 2.3. Chemicals and Drugs

Streptozotocin (STZ) and Gabapentin have been bought from Sigma–Aldrich (St. Louis, USA). All other drugs and chemicals used in the study were of standard quality and of analytical grade, which includes Citric acid (Fisher Scientific), Trisodium citrate (Fisher Scientific), Sodium dihydrogen phosphate (HiMedia Laboratories Pvt. Ltd.), Disodium hydrogen phosphate (HiMedia Laboratories Pvt. Ltd.), Pentobarbital (Kamron Laboratories Ltd., Gujarat, India), Xylazine (Troikaa Pharmaceuticals Ltd.), Ketamine (Troikaa Pharmaceuticals Ltd.), Disodium-EDTA (Fisher Scientific), Isophane insulin injection IP (huminsulin® 30/70) (Eli Lilly and Company Pvt. Ltd, India), Tropicamide eye drops (Sunways (I.) P. Ltd, Mumbai, India), and formaldehyde (Qualigens Fine Chemicals, Mumbai, India). The various standard ELISA as well as other biochemical kits used included Rat Glycated Hemoglobin (KINESISDx), Rat IL-1 *β* (KRISHGEN Biosystems), Rat TNF-*α* (KRISHGEN Biosystems), Rat VEGF ELISA (KRISHGEN Biosystems), Rat PKC-*β* ELISA (KINESISDx), Rat Lipid Peroxide (LPO) ELISA (KINESISDx), Superoxide Dismutase (SOD) Colorimetric Activity Kit (ARBOR assays), and Catalase Colorimetric Activity Kit (ARBOR assays).

### 2.4. Diabetes Induction

By injecting streptozotocin (STZ) at an intraperitoneal dosage of 45 mg/kg (prepared in 0.1 M citrate buffer at a pH of 4.5) into Wistar rats fasted overnight, diabetes was induced. Blood levels of glucose were analyzed initially at 0 time and 48 hours later following the injection of vehicle or streptozotocin. The diabetic animals were considered to be included in the experiment if their blood glucose levels exceeded 300 mg/dl. From the literature study, in order to decrease the mortality in diabetic rats, 1 unit of huminsulin was administered if the blood glucose levels are greater than 450–500 mg/dl, and if they are greater than 600 mg/dl rats, 2 units of huminsulin via subcutaneous route was administered [12].

### 2.5. Design of Experiment

Forty-eight (48) rats had been grouped into eight groupings at random. In group I (Normal control), rats were given saline orally, group II comprised of diabetes rats representing the diabetic group known as diabetic control group, in group III (*Tridax procumbens* per se group), normal rats were given *Tridax procumbens* daily for 28 days, and group IV was Gabapentin-treated diabetic rats who received daily Gabapentin (50 mg/kg, p.o); min before administration. The Gabapentin dose has been selected, referring to an earlier published literature. The experimental design and grouping of animals are summarized as followa:

- Group I: Normal control.
- Group II: Positive control (Diabetic).
- Group III: TP per se group.
- Group IV: Gabapentin (50 mg/kg body weight).
- Group V: TP 250 mg/kg.
- Group VI: TP 375 mg/kg.
- Group VII: TP 500 mg/kg.
- Group VIII: Standard drug (Gabapentin) + TP 500 mg/kg

### 2.6. General Parameters

During the progression of the study, overnight fasted rats were engaged for blood collection initially at week 0 and thereafter at 48 hrs, 1st week, 2nd week, and 3rd week in each group. The tails of the rats were pricked with a syringe, and blood glucose was measured using an electronic glucometer and glucose test strips [13]. During the 4th week of observation, a blood sample varying from 0.5 ml to 1 ml was stored from the retroorbital technique for the estimation of glycated hemoglobin (HbA_1c_) [13]. Before starting the study (baseline value), and 48 hours after STZ administration, the bodyweight was noted. Furthermore, bodyweight of control, diabetic rats, and treated rats was determined from week 1 to week 4 [13]. After acclimatization for a day, we collected the urine samples from animals in individual urine collection cages. From day 0 onward, urine samples were collected from the control, diabetic control, and treatment groups every week to establish baseline data [13].

### 2.7. Biochemical Estimation

In the next step, all rats were decapitated, and both sciatic nerves were carefully excised and rinsed in ice-cold saline. All sciatic nerve samples collected were homogenized in buffer of phosphate &put at a temperature of −80°C for subsequent investigation and estimations of various biochemical markers including catalase (CAT), thiobarbituric acid reactive substances (TBARS), superoxide dismutase (SOD), interleukin-1*β* (IL-1 *β*), tumor necrosis factor-alpha (TNF-*α*), and protein kinase C (PKC-*β*) and vascular endothelial growth factor (VEGF).

#### 2.7.1. Angiogenic and Inflammatory Parameters

By employing Enzyme Linked-Immuno-Sorbent Assay (ELISA), VEGF levels and PKC activity were determined. ELISA kits were employed to estimate the IL-1*β* & TNF-*α* levels as previously described in a previous article [14].

#### 2.7.2. Oxidative Stress Parameters

The amount of thiobarbituric acid reactive substances (TBARS) was estimated using the previously described method. A previously described method was used to determine the catalase levels in sciatic nerve homogenate [15]. As was done previously, an earlier method for measuring SOD was also used to appraise levels of SOD [16].

### 2.8. Behavioral Experiments

#### 2.8.1. Cold Allodynia Using Acetone Test

The mid-plantar region of the hind leg of every group of Wistar rats was gently rubbed with acetone drops (50 *μ*L). There were nociceptive pain responses seen in the form of paw licking, rubbing, shaking, and even withdrawal of food in response to cold stimuli. After application of acetone, a digital stopwatch was used to measure the response time (1 minute). Both paws were sampled three times for each reading, and the mean value was considered [17].

#### 2.8.2. Hot Allodynia Using Eddy's Hot Plate

The nociceptive response to TP is estimated through this method [18]. The animals were placed on hot plate, which were put at a constant 55°C temperature for the duration of experiment. As a feedback response as thermal hyperalgesia, paw licking and jumping were taken with the help of digital stopwatches and considered as positive reactions to heat. The animals that exceeded the cutoff time were removed from the hot plate until baseline values were achieved.

#### 2.8.3. Grip Strength Test Using Rota Rod

An accelerating rotarod apparatus was used to assess rats' motor coordination (Ugo Basile, Italy, Model 7750) [19]. Before the experiment, rats were skilled for three consecutive days at a static speed of 20 to 25 rpm (five minutes each day). During the test day, rats were placed contrary to the rotating rod, which began at 4 rotations per minute (rpm) and accelerated gradually up to 20–25 rpm. A decrease in muscle grip indicates relaxation of muscles. An index of muscle relaxation is taken from the variance in drop off time from the rotating rotarod between groups.

#### 2.8.4. Hyperalgesia Using Tail Flick

In this method, the withdrawal of the tail from the heat is taken as an endpoint [20]. In order to prevent damage to the tail using a hot plate at 55°C, 10–12 sec was observed to be cut off. Animals that do not withdraw their tails within 3–5 seconds are rejected from the study. The reaction time was noted after the drug/TP had been administered at 5, 15, 30, and 60 minutes.

### 2.9. Statistical Assessment

Each of the data points has been expressed as a mean + SD. An ANOVA was conducted as a one-way test, followed by a Tukey-Kramer test, which were conducted as *post hoc* tests based on *p* < 0.05 statistical differences between groups. GraphPad Prism software was used in this study (version 7, GraphPad Software, San Diego, USA).

## 3. Results

### 3.1. General Parameters

#### 3.1.1. Influence of TP on Blood Glucose, Body Weight, HbA_1c_, and Urine Volume

Diabetes results in a significant weight loss to 158.73 ± 1.54 (*p* < 0.01) and a steady statistically significant exponential elevation in blood glucose levels as well as urine volume by approximately 565.22 ± 4.72 (*p* < 0.01) and 80.56 ± 1.27 (*p* < 0.01), and correspondingly, in assessment with normal rats (Table 1). Diabetic rats administered with TP (250, 375, and 500 mg/kg) and standard treatment Gabapentin exhibited substantial decline in the blood glucose levels along with the urine volume as well as upsurge in the bodyweight in treatment rats as equated with diabetic rats. Additionally, the combination treatment of highest dose of TP 500 mg/kg with the standard drug, Gabapentin, produced an additive effect in the decreasing the levels of blood glucose levels but produced nonsignificant differences in the urine volume and bodyweight (Tables 2 and 3). Upon completion of the study, the HbA_1c_ levels were estimated for all study groups. A steady, statistically considerable raise in HbA_1c_ amount was seen in diabetic rats. Diabetic Wister rats administered with TP (250, 375, and 500 mg/kg) and standard Gabapentin showed momentous decrease in HbA_1c_ level. Additionally, the combination treatment of highest dose of TP 500 mg/kg with the standard drug, Gabapentin, produced nonsignificant differences in HbA_1c_ concentration as equated to typical and normal Wister rats (Table 4).

### 3.2. Angiogenic Parameters

#### 3.2.1. Effect of TP on Sciatic VEGF Levels and PKC-*β* Activity

Angiogenesis is designated as de novo formation of newer blood vessels followed by growth. The role of angiogenesis has been available in the literature in development of diabetes related complications of nephropathy. Angiogenic indicators were assessed to be high; namely, PKC-*β* and VEGF were augmented in serum STZ-post administration. PKC- *β* is an essential marker in determining angiogenesis. VEGF initiation is thought to occur as a result of PKC- *β* activation under tumorigenesis, according to the outdated view or ischemic conditions that additionally enhance endothelial cells angiogenesis via a paracrine mechanism. Following STZ administration, PKC- *β* levels increased in the serum of Wistar rats, which led to the development of diabetes. Administration of TP 250, 375, and 500 mg/kg and standard Gabapentin showed noteworthy diminution in PKC-*β* levels. Additionally, the combination treatment of highest dose of TP 500 mg/kg with the standard drug, Gabapentin, produced nonsignificant differences in PKC-*β* levels in maintaining the level of PKC-*β* to the levels found in normal rats (Table 5).

VEGF is a potent biological marker entity employed in appraisal of degree of angiogenesis. From the available literature, it can be deduced that the increase in the levels of VEGF is associated in the rat model of diabetes caused by the administration of STZ. TP (250, 375, and 500 mg/kg) administration and standard treatment Gabapentin displayed substantial decrease in the levels of VEGF. Additionally, the combination treatment of highest dose of TP 500 mg/kg with the standard drug, Gabapentin, produced nonsignificant differences in maintaining the level of VEGF to the levels found in normal Wister rats (Table 5).

### 3.3. Inflammatory Parameters

#### 3.3.1. Effect of TP on Sciatic Levels of IL-1*β* and TNF-*α*

The concentrations of IL-1*β* and TNF-*α* were augmented in serum of rat after administration of STZ as toxicant. Diabetic Wister rats treated with TP (250, 375, and 500 mg/kg) accompanied by the standard drug Gabapentin meaningfully drained the levels of mediators of inflammatory cascade such as TNF-*α* and IL-1*β* to normal. The IL-1 *β* levels as proinflammatory cytokine are exceptionally high in circumstances of diabetes associated complications. Administration of TP (250, 375, and 500 mg/kg) and standard treatment Gabapentin displayed a noteworthy decrease in the levels of VEGF. Additionally, in the combination treatment of highest dose of TP 500 mg/kg with the standard drug, Gabapentin, produced nonsignificant differences in maintaining the IL-1 *β* level to the levels found in normal (Table 5).

There are several biomarkers for describing the tissue inflammation grade, including TNF-*α* as the most common one. From the literature evidence, it has been found that the levels of TNF- *α* significantly increase in animal model of diabetes administered with STZ. In the research, TP administration at mg/kg basis (250, 375, and 500) and standard Gabapentin showed a noteworthy decrease in the TNF- *α* levels. Additionally, the combination treatment of highest dose of TP 500 mg/kg with the standard drug, Gabapentin, produced nonsignificant differences in maintaining the level of TNF-*α* to the levels found in normal rats (Table 5).

### 3.4. Assessment of Oxidative Stress Parameters

#### 3.4.1. Effect of *TP* on Sciatic TBARS, CAT, and SOD

The TBARS levels in all groups were measured after four weeks of treatment. The TBARS levels in diabetic controls have significantly increased (8.02 ± 1.20 U/mg protein). Compared to the untreated groups, the TP administered rats experienced a noteworthy reduction in levels as 6.78 ± 0.46, 6.06 ± 0.31, 4.99 ± 0.24, and 4.57 ± 0.28 U/mg protein for TP 250, 375, and 500 mg/kg and Gabapentin + TP 500 mg/kg, respectively (Table 6).

Sciatic nerve homogenates from diabetic control rats were found to have a significant reduction in SOD (1.44 ± 0.39 U/mg protein) compared to normal rats (4.14 ± 0.37 U/mg protein). In disparity, rats in the TP administered group had higher levels of SOD—2.33 ± 0.43, 2.80 ± 0.05, 3.49 ± 0.15, and3.83 ± 0.25 U/mg protein in Group TP 250, 375, and 500 mg/kg and *TP* 500 mg/kg + Gabapentin, respectively (Table 6). In rats treated with TP and Gabapentin, these have shown significant and dose-dependent effects.

In diabetic control group, CAT levels had been considerably diminished in the sciatic nerve homogenate (1.32 ± 0.29 U/mg of protein) when equated to the normal animals (3.33 ± 0.45 U/mg of protein). A noteworthy upsurge in CAT levels has been detected in TP treated rats—1.66 ± 0.20, 1.91 ± 0.11, 2.48 ± 0.21 and 2.70 ± 0.19 U/mg protein in Group *TP* 250, 375, and 500 mg/kg and *TP* 500 mg/kg + Gabapentin, respectively (Table 6). TP and Gabapentin were shown to produce significant and dose-dependent effects in rats.

### 3.5. Behavioral Experiments

#### 3.5.1. Cold Allodynia Using Acetone Test

Table 7 showed the results. Group *TP* 250, 375, and 500 mg/kg and TP 500 mg/kg + Gabapentin have recorded average readings of 5.12 ± 0.57, 4.73 ± 1.09, 4.67 ± 0.18, and 4.45 ± 0.77, respectively, in cold hyperalgesia results equated to week 4 (^*∗∗*^*p* < 0.01). As the diabetes-induced group recorded delayed responses to hyperalgesia, in the diabetic group, longer responses were observed.

#### 3.5.2. Hot Allodynia in Hot Plate Technique (Eddy's Hot Plate)

TP treated rats (250, 375, and 500 mg/kg) and TP 500 mg/kg + Gabapentin have revealed a mean reaction latency of 6.28 ± 0.65, 5.14 ± 1.23, 4.88 ± 0.23, and 4.56 ± 0.78, respectively, on 4 ^th^ week (Table 8). In equation to the results from the diabetic group during 4 th week, the results were suggestively different (^*∗∗*^*p* < 0.01).

#### 3.5.3. Grip Strength Test Using Rota Rod

Neuromuscular coordination logged an augmentation of 96.93 ± 2.62, 106.88 ± 3.03, 116.75 ± 3.26, and 124.65 ± 3.72 for Group TP 250, 375, and 500 mg/kg and TP 500 mg/kg + Gabapentin, respectively (Table 9). In comparison to diabetes control rats, the outcomes from fourth week of experimentation were quite significant. When comparing all the treated groups to the diabetic control groups, all the outcomes had a noteworthy *p* < 0.01 value. The healing and protective effects of TP and Gabapentin were, therefore, highlighted.

#### 3.5.4. Hyperalgesia Using Tail Flick

The hyperalgesia retorts of rats treated with *TP* 250, 375, and 500 mg/kg and TP 500 mg/kg + Gabapentin have improved to 5.98 ± 1.62, 6.28 ± 1.82, 6.39 ± 1.74, and 6.44 ± 1.68, correspondingly on assessment from 4th week findings (Table 10). The findings were significant (*p* < 0.01).

## 4. Discussion

As of now, no treatment schedule other than belligerent and strict glycemic control has proven effective at halting diabetic neuropathy. Recent studies have demonstrated the effectiveness of natural products and their derivative bioactive phytoconstituents against diabetes in animal models and humans. In the study presented here, TP was investigated to determine whether it had protective effects in contrast to experimentally induced diabetic neuropathy (DN) in diabetic animals, building on earlier studies. STZ administration was linked with significant upsurges in blood glucose and concentration of HbA_1c_ in the present investigation, which is reliable and allied with preceding conclusions reported earlier. As an eminent cytotoxin of *β*-cell of pancreatic tissue, STZ diminishes the insulin secretory functionalities and elevates blood levels of glucose by increasing cellular oxidative stress [21, 22].

Diabetic rats also lost weight due to diabetes. A decrease in insulin levels may be causing the accumulation of amino acids as a result of increased tissue protein turnover for metabolic energy. The treatment with TP resulted in diminution of weight loss, blood sugar, and HbA1c levels. Due to its use as a gauge of glycemic control, HbA1c is habitually associated with diabetes complications over time [23]. Many *in vivo* and *in vitro* studies demonstrated antihyperglycemic properties of *TP* [24, 25]. Many mechanisms, including improved insulin discharge and insulin sensitivity, augmented uptake of glucose, and *α*-glucosidase activity inhibition may underlie TP antihyperglycemic actions. Aside from amending key enzymes responsible for glucose homeostasis in liver, TP might also be attributed to decrease glucose production [25]. There is no doubt that prolonged hyperglycemia contributes to deterioration of the nociceptive edge, a reduction of sensory-motor nerve conduction velocity (NCV), and several manifestations of conjured-pain, including cold allodynia, hot allodynia, grip weakness, and hyperalgesia [26].

The neurobehavioral tests performed in the present study designated that STZ injections caused impaired sensory function based on cold allodynia, hot allodynia, grip strength, and hyperalgesia. However, TP amended sensory aberrations and confirmed significant antinociceptive properties, as evidenced by a decline in paw withdrawal threshold and paw withdrawal latency, as well as a reduction in fall and tail flicking time. As a matter of fact, the results also indicated that TP demonstrated potentiation of the antinociceptive effects of Gabapentin as well. In contrast, while TP treatment altered and ameliorated hyperglycemia and partly reduced and inverted neuropathic pain, the combination of TP and Gabapentin exhibited greater reversal of DN development, suggesting the involvement of other mechanisms besides reducing the elevated glucose levels. The increased reactive nitrogen/oxygen species, oxidative stress, and free radicals, generated by metabolic and vascular insults, contributed to progressive damage and dysfunction of nerve fibers in DN. Neuropathy is generally known to be caused by the excess excitability of the afferent nociceptors and the central neurons present in diabetic peripheral nerve impairment consequently of oxidative stress [27]. The polyol pathway may be responsible for increased NADPH consumption in diabetes, while mitochondrial superoxide production, PKC activation, and glucose autoxidation may also contribute to oxidative stress [28, 29]. Moreover, previous reports have revealed that patients with DN have impaired antioxidant defenses [30, 31]. Therefore, antioxidants may assist in treating DN by reducing oxidative stress. TP treatment diminished protein carbonylation, as well as lipid peroxidation, and increased antioxidant actions in the present study. DN may be caused by oxidative damage to the myelinated structure of nerves, which predominantly consists of lipids [31].

TP has been demonstrated to be a powerful scavenger of free radicals, antioxidants, and enzymes (free radical generating) inhibitor. The phenolic and flavonoid phytocomponents present in this plant extract might be accredited to its capability for scavenging free radicals due to its structural characteristics. The phytoconstituents in TP possessed phenolic nuclei as well as structures with unsaturated side chains, which eases the ability to generate a phenoxy radical owing to stabilization by resonance of the molecules [32].

Oxidative stress is a significant factor in DN progression, and it is primarily caused by the oxidation of monosaccharides and proteins [33]. The findings of the present investigation presented an extraordinary elevation of TBARS as biomarker of peroxidation of lipid, in the sciatic nerve homogenate of diabetic Wister rats. Two endogenous antioxidants, SOD and Catalase (CAT), are considered early defenses against free radicals and ROS or RNS. The current research found that SOD and CAT levels in the sciatic homogenate of diabetic rats had been substantially reduced. An imperative antioxidant enzyme that contributes to decomposition of free radicals is the endogenous defense mechanisms, amongst which are antioxidant enzymes (SOD and CAT). Nonetheless, SOD protects biological tissues and environment from extremely responsive superoxide anions (O2^−^) by transforming them into hydrogen peroxide (H_2_O_2_), and hyperglycemia reduces SOD action in the homogenate of sciatic nerve of Wister rats due to nonenzymatic glycosylation [29]. These data are in treaty with findings from this present study, in which diminished SOD and CAT activity were observed in diabetic rats homogenized isolated sciatic nerves. Contrary to this, CAT is crucial in catalyzing the decomposition of harmful H_2_O_2_ to O_2_ and H_2_O. CAT activation is decreased in diabetes, which decreases cellular defense and makes tissues further vulnerable to free radicals. By inhibiting the enzyme activity in diabetic rats, we also found a connection between DN and oxidative stress. Thus, the simultaneous reduction in endogenous antioxidant defenses system renders sciatic nerves more susceptible to hyperglycemia provoked oxidative stress.

An upsurge in the activity of PKC-*β* and levels of VEGF was observed in this current study in diabetic rats induced with STZ. Hyperglycemia causes impaired nerve conduction due to the possible impairment of the PKC- *β* /HuR/VEGF pathway and increased activity of PKC- *β* and VEGF. A substantial upsurge in this enzyme activity was found in diabetic rats' sciatic nerves and erythrocytes [34, 35]. Furthermore, the creation and excretion of VEGF have been described to increase in experimental models of DN. Previous reports have found that exogenous VEGF can improve diabetic patient outcomes, and neuronal survival and maintenance are reliant on these factors. Antioxidant effects of VEGF may be a result of Bcl-2 upregulation, phosphor Akt pathway activation, stimulation of mTOR signaling, and increased activity of endogenous enzymes responsible for scavenging of free radicals [36]. The TP treatment significantly decreased PKC- *β* activity, and there was a decrease in DN and behavioral parameters among STZ-induced diabetic rats with higher VEGF levels in nerve homogenate. This investigation demonstrated that TP reversed elevations in TNF-*α* and IL-1 *β* levels. In reality, cytokines of proinflammatory kind, for instance IL-1 *β* and TNF-*α*, have been proven to perform a noteworthy part in the pathophysiogenesis of neurodegeneration and transmission of pain in diabetic patient [37]. Furthermore, the activity of IL-1*β* and TNF- *α* is upregulated in dorsal root ganglion region as well as peripheral nerves under hyperglycemic conditions [38]. Treatment with TP had revealed to avert biochemical and functional deficits of peripheral nerves in diabetic animals, as indicated by estimated biomarker levels. The antihyperuricemia, antioxidant, and antibacterial activity [39], anti-inflammatory and antiapoptotic actions [40], and antiosteoporotic activity of TP had been well recognized in several disease models. According to previous studies, TP inhibits the expression of proinflammatory cytokines by constricting NF-*κ*B signaling and NLRP3 inflammasome activity [40]. This present investigation demonstrated that TP enhanced functionality of peripheral nerves in diabetic rats through improved control of glycemic status, suppression of neuroinflammation, oxidative stress, and inhibition of hallmark biomarkers. Despite this, the combination therapy improved nociception and nerve conduction velocities by modulating the angiogenic, inflammatory, and oxidative stress markers more than the individual therapies and may prove advantageous when treating diabetic patients with peripheral neuropathy.

## 5. Conclusion

Based on the findings, *Tridax procumbens* (TP) offered a novel and promising complementary approach to the treatment of diabetes, bringing potential benefits to diabetes neuropathy management. Human subjects can be included in future studies in order to determine what dose of TP extract provides the best control of blood glucose besides optimum management of diabetic neuropathy. TP extract alone for mechanistic studies would validate current findings and allow for detailed phytochemical screening and fractionation for bioactivity-guided assays. In order to determine TP effect on glucose uptake in skeletal muscle cells, independent of insulin signaling, more research is needed. Moreover, future studies can be designed to investigate in more detail the phytochemical composition of the present extract. Clinical trials need to be designed to study the observed therapeutic effect of the plant in relevant human population. The present investigation provided evidence that TP could be beneficial in managing diabetes through improved peripheral nervous health as well as neuroprotective effect, while offering a mechanistic explanation for the traditionally attributed antidiabetic and anti-inflammatory actions.

## Figures and Tables

**Table 1:** Impact of TP on blood level of glucose throughout antidiabetic study model (postgeneration of diabetes).

	Normal control	Disease control	*TP* per se	Standard (Gabapentin)	T.P. 250 mg	*T.P.* 375 mg	*T.P.* 500 mg	*T.P.* 500 mg + Gabapentin
Week 0	225.72 ± 2.51	229.22 ± 4.82	223.26 ± 5.26	220.23 ± 5.24	221.33 ± 4.33	227.75 ± 1.51	211.24 ± 2.61	230.15 ± 4.81
Week 1	234.51 ± 2.51	211.33 ± 1.73	196.26 ± 3.15	205.21 ± 3.13	185.43 ± 2.73^+^	187.33 ± 8.52^*∗*^^,+^	190.05 ± 4.47^*∗*^^,++,a^	202.23 ± 4.51^*∗∗*^^,++,a^
Week 2	229.25 ± 3.16	183.43 ± 1.9	189.6 ± 5.61	187.56 ± 5.6	182.18 ± 3.71	191.85 ± 8.31^*∗*^	197.32 ± 2.81^*∗∗*^^,++,a^	203.61 ± 3.42^*∗∗*^^,++,aa,b^
Week 3	245.72 ± 0.71	173.88 ± 1.01	191.54 ± 6.14	170.51 ± 6.11^*∗∗*^	176.23 ± 4.13^+^	194.25 ± 7.71^*∗∗*^	204.23 ± 6.62^*∗∗*^^,++,a^	224.23 ± 5.3^*∗∗*^^,++,aa,b^
Week 4	257.16 ± 1.84	158.73 ± 1.54^##^	201.36 ± 5.22	167.33 ± 5.24	164.24 ± 3.11^##,+^	178.83 ± 6.7^##,^^*∗∗*^^,+^	212.72 ± 5.14^#,^^*∗∗*^^,++,a^	256.03 ± 4.64^*∗∗*^^,++,aa,b^

**Table 2:** Impact of TP on the body weight.

	Normal control	Diabetic control	Gabapentin 50 mg/kg	*T.P.* 250 mg	*T.P.* 375 mg	*T.P.* 500 mg	*T.P.* 500 mg/kg + Gabapentin
0 Week	91.65 ± 2.84	97.60 ± 3.22	99.09 ± 2.11	100.14 ± 2.51	97.93 ± 1.74	97.36 ± 3.64	96.17 ± 2.84
48 hours	93.28 ± 2.66	524.59 ± 3.51	554.13 ± 2.73	552.64 ± 2.61	547.94 ± 10.34	549.94 ± 3.24	545.56 ± 7.64
Week 1	94.73 ± 1.75	542.92 ± 2.56	515.16 ± 1.84^*∗∗∗*^	529.53 ± 2.81 ^*∗∗*^^,+^	511.74 ± 5.44 ^*∗∗*^^,+^	497.53 ± 3.74 ^*∗∗*^^,++,aa^	485.25 ± 6.28^*∗∗*^^,+,aa,bb^
Week 2	93.27 ± 2.25	551.35 ± 3.12	490.12 ± 2.55^*∗∗*^	501.37 ± 2.71 ^*∗∗*^	487.25 ± 6.44 ^*∗∗*^^,+,a^	480.46 ± 3.86 ^*∗∗∗*^^,++,aa^	415.74 ± 4.84^*∗∗*^^,++,aa,bb^
Week 3	92.29 ± 2.37	554.82 ± 3.13	455.36 ± 1.94^*∗∗*^	477.86 ± 2.81 ^*∗∗*^^,+^	457.67 ± 5.24 ^*∗∗*^^,+,aa^	430.84 ± 3.64 ^*∗∗∗*^^,++,aa^	370.73 ± 4.55^*∗∗∗*^^,++,aa,bb^
Week 4	90.92 ± 1.18	565.22 ± 4.72^###^	418.61 ± 1.58^##,^^*∗∗∗*^	420.64 ± 2.71^###,^^*∗∗∗*^^,+^	397.67 ± 5.75^###,^^*∗∗∗*^^,+,aa^	381.18 ± 3.27^###,^^*∗∗∗*^^,+++,aa^	304.41 ± 4.55^*∗∗∗*^^,++,aaa,bb^

**Table 3:** Impact of TP on the volume of urine postinduction of diabetes in Wistar albino rats.

	Normalcontrol	Diabeticcontrol	Standard(Gabapentin)	*T.P.* 250 mg/kg	*T.P.* 375 mg/kg	*T.P.* 500 mg/kg	*T.P.* 500 mg/kg + Gabapentin
0 Week	13.61 ± 0.23	12.96 ± 0.37	14.39 ± 0.26	13.76 ± 0.24	14.66 ± 0.17	14.84 ± 0.36	13.16 ± 0.38
Week 1	13.78 ± 0.38	54.23 ± 1.33	49.47 ± 1.22^*∗∗*^	64.2 ± 0.44^*∗∗*^^,++^	61.29 ± 2.76^*∗∗*^^,++^	56.16 ± 0.91^*∗∗*^^,+,a^	47.78 ± 2.85^*∗∗*^^,a^
Week 2	14.03 ± 0.48	71.53 ± 1.52	46.58 ± 0.51^*∗∗*^	56.44 ± 0.65^*∗∗*^^,++^	54.21 ± 0.87^*∗∗*^^,+,a^	50.22 ± 0.47^*∗∗*^^,+,a^	41.25 ± 0.69^*∗∗*^^,+,aa^
Week 3	14.06 ± 0.46	74.47 ± 1.34	43.36 ± 0.47^*∗∗*^	54.24 ± 0.44^*∗∗*^^,++^	51.58 ± 0.34^*∗∗*^^,+,a^	44.14 ± 0.33^*∗∗*^^,a^	35.84 ± 0.58^*∗∗*^^,+,aa^
Week 4	15.75 ± 0.46	80.56 ± 1.27^###^	39.23 ± 0.47^##,^^*∗∗*^	56.35 ± 0.37^###,^^*∗∗*^^,+^	47.17 ± 0.21^##,^^*∗∗∗*^^,a^	36.85 ± 0.32^#,^^*∗∗∗*^^,a^	27.68 ± 0.32^#,^^*∗∗∗*^^,++,aa,b^

**Table 4:** Effect of TP on the glycated hemoglobin (HbA1c) levels postinduction of diabetes in Wistar albino rats at week 4.

Groups	Glycated haemoglobin (HbA1c)
Normal control	3.55 ± 0.49
Disease control	6.30 ± 0.33^##^
TP per se	3.53 ± 0.39^*∗∗*^
Standard (Gabapentin)	3.57 ± 0.38^*∗∗*^
TP 250 mg	5.57 ± 0.19^##,^^*∗∗*^^,aa^
TP 375 mg	4.45 ± 0.19^##,^^*∗∗*^^,a,b^
TP 500 mg	3.72 ± 0.27^*∗∗*^^,bb,c^
TP 500 mg + Gabapentin	3.55 ± 0.16^*∗∗*^^,bb,c^

**Table 5:** Impact of TP on endogenous angiogenic and inflammatory biomarkers in rats.

Groups	PKC (ng/ml)	VEGF (pg/ml)	IL-1*β* (pg/ml)	TNF-*α* (pg/ml)
Normal control	21.80 ± 1.9	5.670 ± 0.26	23.16 ± 1.21	2.04 ± 0.23
Disease control	76.73 ± 4.81^###^	11.69 ± 0.73^###^	47.85 ± 4.31^##^	5.70 ± 0.52^###^
Standard (Gabapentin)	22.87 ± 1.97^*∗∗∗*^	6.710 ± 0.84^*∗∗∗*^	24.09 ± 1.73^*∗∗*^	2.59 ± 0.31^*∗∗*^
TP per se	22.03 ± 1.42^*∗∗∗*^	5.902 ± 0.47^*∗∗∗*^	26.26 ± 1.88^*∗∗*^	2.39 ± 0.25^*∗∗*^
TP 250 mg	36.46 ± 1.97^##,^^*∗*^^,a^	8.970 ± 0.52^#,aaa^	35.40 ± 2.06^##,aa^	3.76 ± 0.71^##,a^
TP 375 mg	31.46 ± 1.97^#,^^*∗∗*^^,a,b^	8.193 ± 0.28^#,^^*∗*^^,aa,b^	31.61 ± 1.38^#,^^*∗*^^,aa,b^	3.29 ± 0.28^#,^^*∗*^^,b^
TP 500 mg	25.94 ± 2.16^*∗∗∗*^^,bb,c^	7.165 ± 0.51^*∗∗*^^,b,c^	24.93 ± 1.46^*∗∗*^^,bb,c^	2.53 ± 0.24^*∗∗*^^,b^
TP 500 mg + gabapentin	24.01 ± 1.56^*∗∗∗*^^,bb,c^	6.877 ± 0.12^*∗∗*^^,bb,cc^	23.24 ± 0.84^*∗∗*^^,bb,c^	2.19 ± 0.12^*∗∗*^^,b,c^

**Table 6:** Impact of TP on endogenous oxidative stress biomarkers in rats.

Groups	TBARS (U/mg protein)	Catalase (U/mg protein)	SOD (U/mg protein)
Normal control	3.98 ± 0.42	3.33 ± 0.45	4.14 ± 0.37
Disease control	8.02 ± 1.20^###^	1.32 ± 0.29^##^	1.44 ± 0.39^###^
Standard (Gabapentin)	4.94 ± 0.39^*∗∗*^	2.51 ± 0.13^*∗∗*^	3.60 ± 0.53^*∗∗*^
TP per se	5.27 ± 0.31	2.83 ± 0.25	3.76 ± 0.29
TP 250 mg	6.78 ± 0.46^#,^^*∗∗*^^,aa^	1.66 ± 0.20^#^^,aa^	2.33 ± 0.43^#,^^*∗*^^,aa^
TP 375 mg	6.06 ± 0.31^#,^^*∗∗*^^,a^	1.91 ± 0.11^#,^^*∗*^^,a^	2.80 ± 0.05^#,^^*∗*^^,a^
TP 500 mg	4.99 ± 0.24^*∗∗*^^,b,c^	2.48 ± 0.21^*∗∗*^^,bb,c^	3.49 ± 0.15^*∗∗*^^,b^
TP 500 mg + Gabapentin	4.57 ± 0.28^*∗∗*^^,bb,cc^	2.70 ± 0.19^*∗∗*^^,bb,cc^	3.83 ± 0.25^*∗∗*^^,bb,c^

**Table 7:** Impact of TP on Cold hyperalgesia: acetone drop test in Wistar albino rats.

	0 Week	1 Week	2 Week	3 Week	4 Week
Normal control	4.4 ± 0.22	4.43 ± 0.05	4.63 ± 1.21	4.61 ± 1.72	4.53 ± 0.62
Disease control	4.18 ± 0.83	5.95 ± 1.02	6.86 ± 1.27	7.85 ± 1.62	9.75 ± 1.22^##^
Standard (Gabapentin)	4.63 ± 1.02	5.43 ± 1.1^*∗*^	5.13 ± 1.32^*∗∗*^	4.83 ± 1.17^*∗∗*^	4.4 ± 1.15^*∗∗∗*^
TP 250 mg/kg	4.33 ± 0.85	5.93 ± 1.04^a^	5.6 ± 1.05^*∗∗*^^,a^	5.18 ± 0.98^*∗∗*^^,a^	5.12 ± 0.57^##,^^*∗∗*^^,aa^
TP 375 mg/kg	4.53 ± 0.71	5.89 ± 1.08^a^	5.69 ± 0.98^*∗∗*^^,aa^	5.23 ± 1.11^*∗∗*^^,a^	4.73 ± 1.09^##,^^*∗∗*^^,a^
TP 500 mg/kg	4.73 ± 1.06	5.58 ± 0.99^*∗*^^,a^	5.34 ± 1.03^*∗∗*^^,a^	5.08 ± 1.01^*∗∗*^	4.67 ± 0.18^*∗∗*^
TP 500 mg + Gabapentin	4.69 ± 0.86	5.47 ± 1.02^*∗*^^,bb^	5.14 ± 1.01^*∗*^^,bb^	5.03 ± 0.87^*∗∗*^^,b^	4.45 ± 0.77^*∗∗*^^,bb^

**Table 8:** Impact of TP on Thermal Hyperalgesia: Eddy's hot plate test to assess diabetic neuropathy in Wistar albino rats.

	0 Week	1 Week	2 Week	3 Week	4 Week
Normal control	4.29 ± 0.21	4.43 ± 0.051	4.63 ± 1.22	4.64 ± 1.82	4.45 ± 0.51
Disease control	4.5 ± 0.51	5.99 ± 1.71	6.68 ± 1.14	7.69 ± 1.81	9.73 ± 1.52^##^
Standard (Gabapentin)	4.92 ± 1.1	5.53 ± 1.01^*∗*^	5.16 ± 2.51^*∗∗*^	4.99 ± 1.01^*∗∗*^	4.63 ± 1.52^*∗∗∗*^
TP 250 mg/kg	4.92 ± 1.11	5.96 ± 1.63^a^	6.63 ± 1.61^*∗∗*^^,a^	6.36 ± 0.51^*∗∗*^^,a^	6.28 ± 0.65^##,^^*∗∗*^^,aa^
TP 375 mg/kg	4.82 ± 1.73	5.96 ± 1.61^*∗*^^,a^	5.74 ± 2.09^*∗∗*^^,aa^	5.34 ± 1.81^*∗∗*^^,a^	5.14 ± 1.23^##,^^*∗∗*^^,a^
TP 500 mg/kg	4.88 ± 1.51	5.54 ± 0.5	5.38 ± 1.76^*∗∗*^^,a^	5.11 ± 1.56^*∗∗*^	4.88 ± 0.23^*∗∗*^
TP 500 mg + Gabapentin	4.94 ± 1.71	5.45 ± 1.34^*∗*^^,bb^	5.22 ± 1.21^*∗*^^,bb^	5.12 ± 0.92^*∗∗*^^,b^	4.56 ± 0.78^*∗∗*^^,bb^

**Table 9:** Impact of TP on Grip strength: Rota rod test to assess muscle grip strength to evaluate DPN in Wistar rats.

	0 Week	1 Week	2 Week	3 Week	4 Week
Normal control	133.21 ± 2.32	137.32 ± 2.62	133.85 ± 3.32	139.73 ± 3.92	138.25 ± 3.42
Disease control	133.51 ± 2.41	79.88 ± 4.71^#^	63.55 ± 2.14^##^	46.45 ± 2.94^##^	36.58 ± 2.41^##^
Standard (Gabapentin)	135.85 ± 3.12	85.35 ± 1.14^*∗*^	94.1 ± 2.39^*∗∗*^	109.95 ± 3.23^*∗∗*^	123.38 ± 2.63^*∗∗∗*^
TP 250 mg/kg	138.85 ± 2.65	86.75 ± 2.65^a^	80.65 ± 2.23^*∗∗*^^,a^	85.25 ± 3.3^*∗∗*^^,a^	96.93 ± 2.62^##,^^*∗∗*^^,aa^
TP 375 mg/kg	138.73 ± 1.39	84.8 ± 2.61^*∗*^^,a^	90.72 ± 3.11^*∗∗*^^,aa^	90.15 ± 2.8^*∗∗*^^,a^	106.88 ± 3.03^##,^^*∗∗*^^,a^
TP 500 mg/kg	136.75 ± 2.71	91.55 ± 3.52	96.32 ± 3.91^*∗∗*^^,a^	99.09 ± 2.78^*∗∗*^	116.75 ± 3.26^*∗∗*^
TP 500 mg + Gabapentin	137.81 ± 2.91	85.45 ± 3.29^*∗*^^,bb^	93.15 ± 4.32^*∗*^^,bb^	108.01 ± 4.18^*∗∗*^^,b^	124.65 ± 3.72^*∗∗*^^,bb^

**Table 10:** Impact of TP on the thermal hyperalgesia in tail flick technique to appraise the neuropathic pain in Wistar rats.

	0 Week	1 Week	2 Week	3 Week	4 Week
Normal control	6.29 ± 0.21	6.49 ± 1.33	5.91 ± 1.24	5.69 ± 0.99	6.55 ± 1.42
Disease control	6.56 ± 2.51	5.35 ± 1.35^#^	4.44 ± 1.25^##^	4.01 ± 1.92^##^	3.31 ± 1.52^##^
Standard (Gabapentin)	6.51 ± 1.05	5.48 ± 1.1^*∗*^	5.64 ± 1.51^*∗∗*^	5.91 ± 1.11^*∗∗*^	6.42 ± 1.41^*∗∗∗*^
TP 250 mg/kg	6.46 ± 1.08	5.38 ± 1.63^a^	5.45 ± 1.11^*∗∗*^^,a^	5.64 ± 1.53^*∗∗*^^,a^	5.98 ± 1.62^##,^^*∗∗*^^,aa^
TP 375 mg/kg	6.67 ± 1.21	5.41 ± 1.76^*∗*^^,a^	5.56 ± 1.39^*∗∗*^^,aa^	5.71 ± 2.09^*∗∗*^^,a^	6.28 ± 1.82^##,^^*∗∗*^^,a^
TP 500 mg/kg	6.36 ± 1.51	5.42 ± 1.43	5.68 ± 1.86^*∗∗*^^,a^	5.83 ± 1.68^*∗∗*^	6.39 ± 1.74^*∗∗*^
TP 500 mg + Gabapentin	6.59 ± 1.51	5.58 ± 1.55^*∗*^^,b^	5.69 ± 1.82^*∗*^^,b^	5.89 ± 1.42^*∗∗*^^,b^	6.44 ± 1.68^*∗∗*^^,b^

## Data Availability

The data used or analyzed during the study are available from the corresponding authors.
